# Real-world outcomes of [^177^Lu]Lu-PSMA-I&T in [^18^F]FDG-positive metastatic castration-resistant prostate cancer: factors related to response and survival

**DOI:** 10.1007/s00259-026-07972-6

**Published:** 2026-06-06

**Authors:** Giulia Santo, Gianpaolo di Santo, Ariane Kronthaler, Mohsen Farsad, Lisa Maria Rossetti, Peter Lehmann, Fabian Scherbauer, Francesco Cicone, Irene J. Virgolini

**Affiliations:** 1 Department of Nuclear Medicine, Medical University of Innsbruck, Anichstrasse 35, Innsbruck, 6020 Austria; 2 Nuclear Medicine Unit, Department of Experimental and Clinical Medicine, ‘‘Magna Graecia’’ University of Catanzaro, Catanzaro, Italy; 3 Department of Nuclear Medicine, Central Hospital of Bolzano, Bozen, Italy

**Keywords:** Radioligand therapy, mCRPC, FDG, PSMA, Treatment response

## Abstract

**Background:**

Little is known about predictors of response to radionuclide therapy with PSMA-ligands in patients with [^18^F]FDG-positive metastatic castration-resistant prostate cancer (mCRPC). We assessed the correlation between baseline characteristics, including dual tracer PET parameters, and response to [^177^Lu]Lu-PSMA-I&T in a cohort of patients with [^18^F]FDG-positive mCRPC. Prognostic factors related to progression-free survival (PFS) and overall survival (OS) were also investigated.

**Methods:**

mCRPC patients who underwent [^68^Ga]Ga-PSMA-11 and [^18^F]FDG PET/CT prior to [^177^Lu]Lu-PSMA-I&T were retrospectively evaluated. Only [^18^F]FDG-positive patients were included in the analysis. A semi-automatic segmentation tool was applied to measure the whole-body maximum standardized uptake value (SUV_max_), mean standardized uptake value (SUV_mean_), metabolic tumor volume (MTV), and total lesion uptake (TLU) on both PET/CTs. PSA response was defined as ≥ 30% and ≥ 50% decline. Imaging response, assessed two months after the last cycle, was defined according to RECIST 1.1 and/or PSMA PET progression (PPP) criteria. Clinical, biochemical, and imaging-based factors were correlated to response to treatment, PFS, and OS.

**Results:**

Twenty-five [^18^F]FDG-positive patients who received [^177^Lu]Lu-PSMA-I&T were included. PSA30 and PSA50 responses were achieved in 11/25 (44%) and in 9/25 (36%) patients, respectively. In the univariate analysis [^68^Ga]Ga-PSMA-11 SUV_mean_ (*p* = 0.022) and [^68^Ga]Ga-PSMA/[^18^F]FDG SUV_mean_ ratio (*p* = 0.021) were significantly associated with PSA30 response. [^68^Ga]Ga-PSMA-11 SUV_mean_ was also significant in the multivariate model. ROC analysis indicated an optimal [^68^Ga]Ga-PSMA-11 SUV_mean_ cutoff value of 10.23 (AUC = 0.741), and a best discriminating [^68^Ga]Ga-PSMA/[^18^F]FDG SUV_mean_ cutoff ratio of 2.08 (AUC = 0.727). In the univariate analysis for radiological PFS (rPFS), high ALP (*p* = 0.023), [^68^Ga]Ga-PSMA-11 MTV (*p* = 0.011), [^68^Ga]Ga-PSMA-11 TLU (*p* = 0.015), and [^18^F]FDG MTV (*p* = 0.013) were significantly associated with shorter rPFS. [^68^Ga]Ga-PSMA-11 MTV showed a borderline association with rPFS in the multivariate model (*p* = 0.061). Hemoglobin levels (*p* = 0.008), ALP (*p* = 0.018) and PSA (*p* = 0.023) values before treatment were associated with OS, as well as [^68^Ga]Ga-PSMA-11 MTV (*p* = 0.003), [^68^Ga]Ga-PSMA-11 TLU (*p* = 0.025), and [^18^F]FDG MTV (*p* = 0.024). However, no independent predictors of OS were identified in the multivariate analysis.

**Conclusions:**

Our preliminary results suggest that a whole-body [^68^Ga]Ga-PSMA-11 SUV_mean_ higher than 10 and a [^68^Ga]Ga-PSMA-11/[^18^F]FDG SUV_mean_ ratio > 2 may serve as useful predictors for identifying patients likely to achieve at least a 30% reduction in PSA levels, even among [^18^F]FDG-positive mCRPC patients. Furthermore, our findings support the prognostic significance of tumor burden either calculated by imaging-based volumetric parameters or by known biochemical markers of disease.

**Supplementary Information:**

The online version contains supplementary material available at https://doi.org/10.1007/s00259-026-07972-6.

## Introduction

Over the past decade, prostate-specific membrane antigen (PSMA) radioligand therapy (PRLT) has consistently demonstrated its efficacy in patients with metastatic castration-resistant prostate cancer (mCRPC) in prospective, randomized clinical trials [1, 2], ultimately leading to regulatory approval by both the U.S. Food and Drug Administration (FDA) and the European Medicines Agency (EMA) in 2022. Today, PRLT is incorporated into major oncology guidelines as a third-line treatment option for patients with progressive mCRPC previously treated with androgen receptor pathway inhibitors and taxane-based chemotherapy [3, 4].

Currently, PSMA expression assessed by uptake on pre-treatment PSMA-targeted positron emission tomography/computed tomography (PET/CT) remains the key eligibility criteria for PRLT, alongside confirmation of adequate hematologic and renal function [5]. However, a considerable proportion of patients do not respond to treatment, and validated biomarkers of response are still lacking. Beyond clinical, biochemical, and molecular factors known to influence outcomes in PCa, quantitative and qualitative PET imaging features have emerged as promising biomarkers to guide patient selection [6, 7].

In this context, [^18^F]FDG PET/CT has prognostic significance [2, 8, 9], but its role in patient selection for PRLT remains highly debated. The rationale for incorporating [^18^F]FDG PET lies in its ability to assess tumor aggressiveness, especially in advanced stages [10]. Although early-stage PCa primarily relies on lipid metabolism rather than aerobic glycolysis, disease progression and accumulating mutational events can drive a metabolic shift toward a Warburg-like phenotype with increased glucose uptake [11]. However, patients with [^18^F]FDG-positive disease have been heterogeneously included in clinical trials [2, 12] or in smaller previous studies [8, 9] and data on real-world clinical experience are missing.

The aim of this retrospective study was to evaluate the association between dual-tracer imaging parameters, baseline characteristics, and response to [^177^Lu]Lu-PSMA-I&T in a cohort of [^18^F]FDG-positive mCRPC patients. In addition, prognostic factors related to progression-free survival (PFS) and overall survival (OS) were evaluated.

## Methods

### Patients

Patients who underwent [^68^Ga]Ga-PSMA-11 and [^18^F]FDG PET/CT before treatment with [^177^Lu]Lu-PSMA-I&T at the Department of Nuclear Medicine of the Medical University of Innsbruck (AT) were included in this retrospective study. All patients included were mCRPC patients and received [^177^Lu]Lu-PSMA-I&T under compassionate use in accordance with the principles of the 1964 Declaration of Helsinki and its subsequent amendments [13]. [^177^Lu]Lu-PSMA-I&T was prepared according to the Austrian Medicinal Products Act AMG § 8 and § 62 and all regulations of the Austrian Agency for Radiation Protection were observed [14, 15]. The study was approved by the ethical committee of the Medical University of Innsbruck (approval no. 1296/2024).

According to the Austrian consensus report on PRLT [16], a [^18^F]FDG PET/CT before the start of PRLT was performed in addition to the [^68^Ga]Ga-PSMA-11 PET/CT scan in highly pretreated mCRPC patients with the aim of identifying mismatch lesions and/or highly dedifferentiated disease ([^18^F]FDG uptake > [^68^Ga]Ga-PSMA-11 uptake) for preliminary risks assessment. Patients fulfilling the following criteria were included in the study analysis: (i) progressive mCRPC treated with at least one cycle of [^177^Lu]Lu-PSMA-I&T RLT; (ii) at least one [^18^F]FDG-positive lesion before the start of PRLT. Patients who performed [^18^F]FDG PET/CT after treatment initiation were excluded from the analysis. The following clinical data were retrospectively extracted from medical records: age, ISUP grade, date of initial diagnosis, time from diagnosis to first PRLT cycle, previous treatments and current medication for PCa, site of metastases before first PRLT cycle. Laboratory tests were carried out before every treatment infusion and at every follow-up visit after treatment discontinuation, when available. Lab examination included blood cell count, renal function (i.e., creatinine, eGFR), liver function (i.e., AST, ALT, total bilirubin), albumin, alkaline phosphatase (ALP), and PSA.

### Treatment schedule

Sufficient hydration was ensured by intravenous infusion (i.e., 1000 ml of 0.9% saline at a rate of 300 ml/h) starting 30 min in advance and 2 h following administration. Prophylactic antiemetic therapy (i.e., ondansetron 20 mg) was given 20 min before and 3–4 h after treatment administration. No specific measures to minimize xerostomia were adopted. [^177^Lu]Lu-PSMA-I&T was administered intravenously over 1 h by a dedicated peristaltic infusion pump system (flow 100 ml/h, 100 ml). PSMA-I&T was obtained from piCHEM (Raaba-Grambach, Austria). [^177^Lu]LuCl_3_ was obtained in no-carrier-added (n.c.a.) quality (EndolucinBeta^®^, ITM Medical Isotopes GmbH, Garching, Germany). [^177^Lu]Lu-PSMA-I&T was prepared as previously described [17]. The final product met the specifications of the European Pharmacopoeia [18]. According to the Austrian Radiation Protection laws, all patients were treated as in-patients at the Nuclear Medicine ward and were discharged 48 h post-injection. The treatment schedule included the intravenous injection of [^177^Lu]Lu-PSMA-I&T with a median activity of 7.6 GBq (range: 6.7–8.0 GBq) per cycle, 5 to 7 weeks apart for up to 6 cycles.

### Imaging protocol

All scans were performed on a dedicated PET/CT system with time-of-flight capability (Discovery MI; GE Healthcare). A whole-body PET scan, covering the region from the skull vertex to the upper thighs, was acquired in three-dimensional mode 1-hour post-injection with an axial field of view of 20 cm and an emission time of 2 min per bed position. A low-dose CT scan was used for attenuation correction of the PET emission data. All PET images were corrected for random events, scatter, and decay. Image reconstruction was performed using the VUE Point FX iterative reconstruction method (GE Healthcare) without a z-axis filter, in combination with the Q.Clear software package (GE Healthcare), a fully convergent iterative reconstruction method with noise control. [^68^Ga]Ga-PSMA-11 and [^18^F]FDG PET/CT scans were performed within a median interval of 37 days (range: 27–55 days) from each other.

### Image analysis

[^68^Ga]Ga-PSMA-11 and [^18^F]FDG PET/CT scans were reviewed by two independent board-certified nuclear medicine physicians blinded to clinical data. Affinity 5.0.1 software within the Hermia platform (Hermes Medical Solutions, Stockholm, Sweden) was used for the simultaneous visualization of PET, CT, and fused imaging data in axial, coronal, and sagittal slices, and for quantification. A semi-automatic segmentation tool was applied to measure the whole-body maximum standardized uptake value (SUV_max_), mean standardized uptake value (SUV_mean_), metabolic tumor volume (MTV), and total lesion uptake (TLU) on both PET/CT scans, and the ratio between [^68^Ga]Ga-PSMA-11 and [^18^F]FDG PET metrics was subsequently calculated. On [^68^Ga]Ga-PSMA-11 PET, whole-body tumor volume was delineated using a SUV threshold of 3 or higher. For segmentation of [^18^F]FDG PET images, a SUV threshold greater than or equal to the liver SUV_mean_ plus 2 standard deviation (SDs) was used, as previously described [12, 19]. The organ segmentation tool implemented in HERMIA – Affinity (version 5.0.1; Hermes Medical Solutions) was used for liver segmentation.

To be defined [^18^F]FDG-positive, patients were required to have at least one macroscopic lesion, highly suspicious for prostate cancer metastasis, exceeding the SUV threshold described above (SUV ≥ liver SUV_mean_ + 2 SDs). Patients who did not fulfill these criteria were considered [^18^F]FDG-negative and excluded from the analysis. The presence of mismatch lesions (FDG+/PSMA-) was recorded and reported.

### Outcome analysis

The response was evaluated as PSA response rate, defined either as the proportion of patients with a PSA reduction of at least 30% (PSA30) or 50% (PSA50) from baseline, respectively [20]. Imaging response was scheduled two months after the last treatment cycle and defined according to RECIST 1.1 on contrast-enhanced CT and/or PSMA PET progression (PPP) criteria based on [^68^Ga]Ga-PSMA-11 [21].

PSA progression free-survival (PSA-PFS) was assessed from the date of first treatment cycle until PSA progression. PSA progression was defined as a ≥ 25% increase in PSA and an absolute increase of 2 ng/mL or more from the nadir, confirmed by a second consecutive value. Where no decline from baseline was documented, PSA progression was defined as a 25% increase from the baseline value along with an increase in absolute value of 2 ng/mL or more after 12 weeks of treatment [1]. Radiological PFS (rPFS) was defined as the time from first treatment cycle to disease progression at follow-up imaging or death. OS was defined from the first cycle of [^177^Lu]Lu-PSMA-I&T until death. Patients who remained event-free were censored at the date of last follow-up.

### Statistical analysis

Statistical analyses were performed using R version 4.5.3 (GUI 1.82 Big Sur Intel build) and GraphPad Prism version 10.6.1 (GraphPad Software, Boston, MA, USA). Microsoft Excel (Microsoft Office 2010) was used to generate waterfall plots of PSA changes. Categorical and continuous variables were analyzed using descriptive statistics. Categorical variables were compared using Pearson’s chi-square test. The distribution of continuous variables was assessed using the Shapiro-Wilk test. Depending on the distribution, parametric or non-parametric tests were applied for comparisons between groups. Mean values of PET metrics derived from [^68^Ga]Ga-PSMA-11 and [^18^F]FDG were compared using paired t-tests. The correlation between [^68^Ga]Ga-PSMA-11 and [^18^F]FDG-derived quantitative parameters was assessed using Pearson’s correlation coefficient (r). Univariate and multivariate penalized Firth logistic regression analyses were performed for each outcome (PSA30 and PSA50) to account for small sample sizes and rare events. Accordingly, variables with p-values < 0.2 were considered for multivariate analysis. Collinearity among variables was assessed using the Variance Inflation Factor (VIF). After excluding highly collinear variables (VIF > 5), the two most significant variables were included in the multivariate model. Receiver operating characteristic (ROC) curve analysis was performed to determine the optimal cut-off value of all significant PET parameters. Survival analysis was performed using the Kaplan-Meier method. Univariable Cox regression analyses were performed to evaluate associations with PSA-PFS, rPFS and OS. Significant independent variables, after excluding collinearity, were selected for inclusion in the multivariate Cox model. A p-value < 0.05 was considered statistically significant.

## Results

**Fig. 1:**
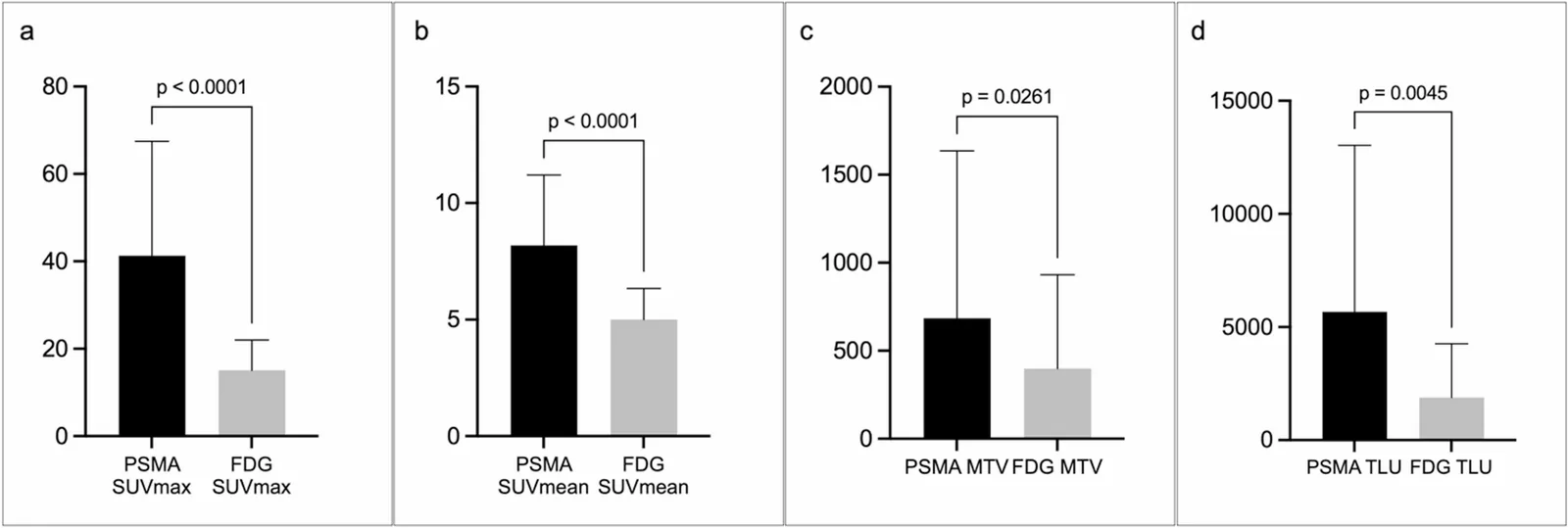
Comparison of PET metrics between [^68^Ga]Ga-PSMA-11 and [^18^F]FDG. [^68^Ga]Ga-PSMA-11 demonstrated significantly higher values than [^18^F]FDG across all parameters, including (**a**) SUV_max_ (41.30 ± 26.16 vs. 15.06 ± 6.94), (**b**) SUV_mean_ (8.18 ± 3.02 vs. 5.03 ± 1.33), (**c**) MTV (685.9 ± 949.9 mL vs. 361.3 ± 400.3 mL), and (**d**) TLU (5681 ± 7350 vs. 1779 ± 2033), with all differences reaching statistical significance. Data are presented as mean ± standard deviation

A total of 34 patients who underwent dual-tracer [^68^Ga]Ga-PSMA-11 and [^18^F]FDG PET/CT scans before treatment with [^177^Lu]Lu-PSMA-I&T between February 2020 and March 2024 were assessed. Nine patients (26%) were classified as [^18^F]FDG-negative and were excluded from the main analysis. Patient characteristics of [^18^F]FDG-negative patients and factors associated with [^18^F]FDG-positivity are described in the supplementary Tables 1 and Fig. 1, respectively. A total of 25 patients met the inclusion criteria and were included in the final analysis. The characteristics of the study population are detailed in Table 1. Median age at the time of the first administration of [^177^Lu]Lu-PSMA-I&T was 69 years (range: 64–77 years) with a median time from PCa diagnosis to PRLT of 80 months (range: 36–148.8 months). At the time of PRLT, 22/25 (88%) patients presented with bone metastases, 18/25 (72%) had lymph node involvement, and 7/25 (28%) patients had visceral metastases. All patients were on androgen deprivation therapy (ADT) at the time of treatment. Twenty patients (80%) had undergone one or more lines of chemotherapy before PRLT.

**Table 1:** Patient characteristics (*n* = 25)

Parameter	Value
Age at C1 (years)	69 (64–77)
ISUP grade 1 2 3 4 5 Missing	3 (12) 2 (8) 1 (4) 10 (40) 6 (24) 3 (12)
Prior treatment Docetaxel Cabazitaxel Enzalutamide History of Ongoing Abiraterone History of Ongoing PARPi ^223^RaCl_2_ Missing	18 (72) 11 (44) 11 (44) 5 (20) 12 (48) 1 (4) 2 (8) 3 (12) 1 (4)
Laboratory data before 1 st PRLT cycle PSA (ng/ml) Hemoglobin (g/L) White cell count (10^9^/L) Platelet count (10^9^/L) Creatinine (mg/dL) Alkaline phosphatase (U/l)	60.5 (0.25–1497) 11.1 (7.8–14.3) 5.8 (3.7–10.1) 230 (62–448) 0.85 (0.61–2.3) 101 (42–3477)
Site of metastases before PRLT Bone Exclusive bone disease Lymph node Exclusive lymph node disease Visceral Liver Lung Peritoneal Brain	22 (88) 5 (20) 18 (72) 3 (12) 7 (28) 5 (20) 3 (12) 3 (12) 2 (8)
For continuous variables median values (25% − 75% percentile) are reported. For categorical variables, number of patients (percent) are reported.	

*C1* 1 st PRLT cycle, *PARPi* poly-ADP-ribose polymerase inhibitors, *PRLT* prostate-specific membrane antigen targeted radioligand therapy, *PSA* prostate specific antigen

Patients received a median of 4 (range: 1–6) cycles of [^177^Lu]Lu-PSMA-I&T with a median cumulative activity of 30.9 GBq (range: 6.7–48.2 GBq).

## Correlation between [^68^Ga]Ga-PSMA-11 and [^18^F]FDG PET metrics

All semiquantitative parameters of [^68^Ga]Ga-PSMA-11 uptake were significantly higher than those of [^18^F]FDG (*paired t-test*, Fig. 1). Mismatch lesions were recorded in 2/25 patients (8%).

There were no significant correlations between [^68^Ga]Ga-PSMA-11 and [^18^F]FDG uptake-based parameters (i.e., SUV_max_ *r* = 0.10 and SUV_mean_ *r* = 0.18, all *p* > 0.40). In contrast, positive correlations were observed between [^68^Ga]Ga-PSMA-11 MTV and [^18^F]FDG MTV (*r* = 0.81, *p* < 0.001), as well as between [^68^Ga]Ga-PSMA-11 TLU and [^18^F]FDG TLU (*r* = 0.66, *p* < 0.001).

## Correlation between patient characteristics and PSA response

### PSA30 response

**Fig. 2:**
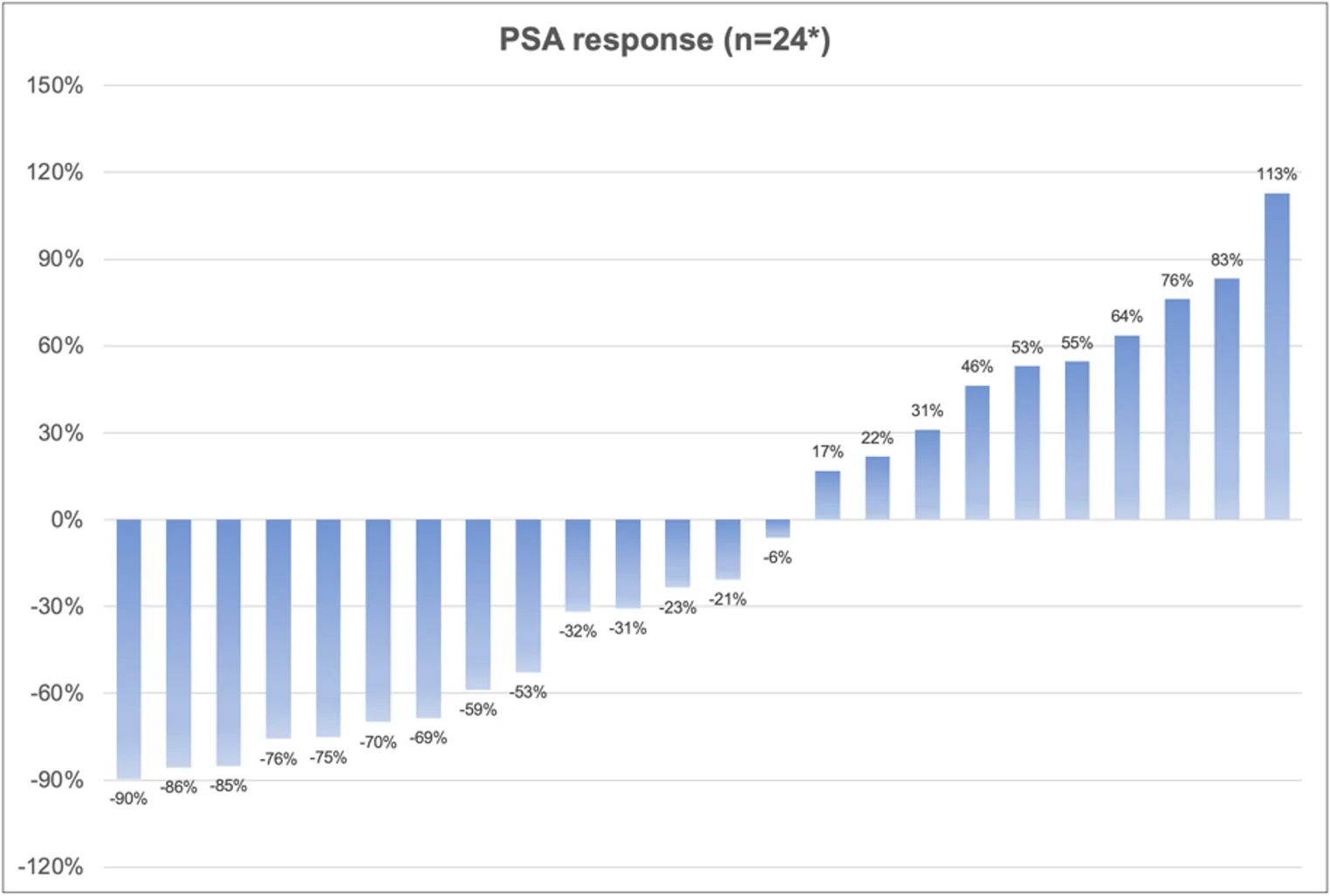
Waterfall plots showing PSA changes in 24 [^18^F]FDG-positive patients who underwent [^177^Lu]Lu-PSMA-I&T. *One patient died after the first cycle

**Fig. 3:**
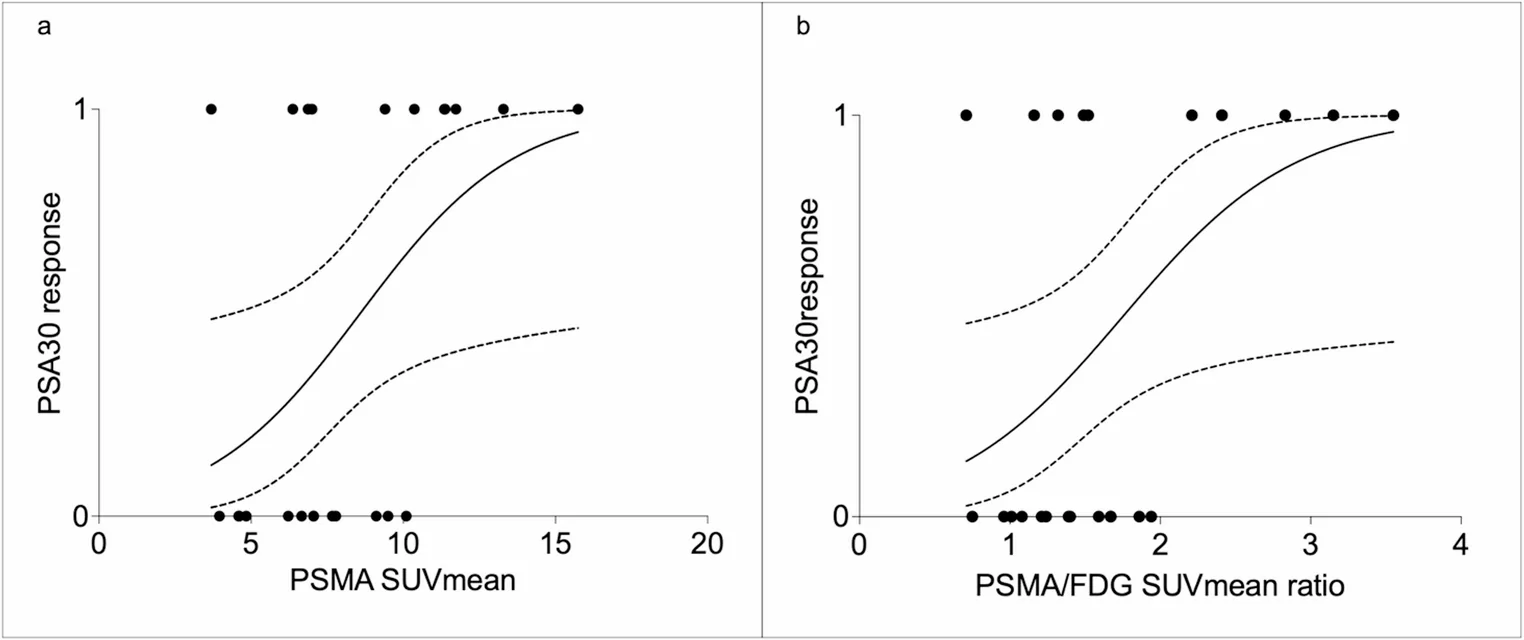
Correlation between (**a**) pre-treatment [^68^Ga]Ga-PSMA SUV_mean_ or (**b**) [^68^Ga]Ga-PSMA/[^18^F]FDG SUV_mean_ ratio and the achievement of a PSA30 response. The figures show that patients with [^68^Ga]Ga-PSMA SUV_mean_ <10 and a [^68^Ga]Ga-PSMA/[^18^F]FDG SUV_mean_ ratio < 2 have a low probability of responding to PRLT

PSA30 response was achieved in 11/25 (44%) [^18^F]FDG-positive patients (Fig. 2), after a median time from the first PRLT administration of 84 days (range: 45–135 days). Firth univariate regression showed a significant association between PSA30 response and whole-body [^68^Ga]Ga-PSMA-11 SUV_mean_ (OR 1.402, 95%CI 1.044–2.095, *p* = 0.022, Fig. 3a) as well as with the [^68^Ga]Ga-PSMA-11/[^18^F]FDG SUV_mean_ ratio (OR 4.265, 95%CI 1.216–27.386, *p* = 0.021, Fig. 3b). [^68^Ga]Ga-PSMA SUV_max_ showed a trend toward a significant correlation with PSA30 response (OR 1.029, 95%CI 0.998–1.068, *p* = 0.066). The multivariate model confirmed whole-body [^68^Ga]Ga-PSMA-11 SUV_mean_ as an independent predictor of PSA30 response (OR 1.493, 95%CI 1.071–2.443, *p* = 0.016). Table 2 shows the results of the regression analysis.

**Table 2:** Univariate and multivariate logistic regression analysis for PSA30 response

Variable	Univariate		Multivariate	
	Odd ratio (95%CI)	*p*	Odd ratio (95%CI)	*p*
Clinical factors				
Time from diagnosis to PRLT	1.001 (0.997–1.018)	0.194		
Bone mets (no vs. yes)	0.456 (0.037–4.016)	0.476		
Lymph node mets (no vs. yes)	1.150 (0.2133–6.564)	0.870		
Visceral mets (no vs. yes)	0.555 (0.080–3.208)	0.514		
Chemotherapy (yes vs. no)	2.111 (0.283–24.775)	0.474		
N° previous lines (< 2 vs. ≥2)	0.760 (0.055–10.508)	0.824		
Biochemical factors				
White cell count (10^9^/L)	1.388 (0.847–2.556)	0.199		
Hemoglobin (g/L)	1.038 (0.986–1.105)	0.159	1.052 (0.992–1.134)	0.090
Platelet count (10^9^/L)	0.997 (0.987–1.008)	0.641		
Alkaline phosphatase (U/L)	1.003 (0.994–1.021)	0.448		
PSA (ng/mL)	0.999 (0.979–1.020)	0.941		
Imaging-based factors				
PSMA SUV_max_	1.029 (0.998–1.068)	0.066		
PSMA SUV_mean_	1.402 (1.044–2.095)	0.022	**1.493 (1.071–2.443)**	**0.016**
PSMA MTV	1.002 (0.993–1.013)	0.554		
PSMA TLU	1.006 (0.993–1.021)	0.339		
FDG SUV_max_	0.975 (0.858–1.086)	0.641		
FDG SUV_mean_	0.994 (0.543–1.755)	0.981		
FDG MTV	1.004 (0.991–1.024)	0.509		
FDG TLU	1.009 (0.979–1.048)	0.534		
PSMA/FDG SUV_max_	1.346 (0.947–2.088)	0.099		
PSMA/FDG SUV_mean_	4.265 (1.216–27.386)	0.021		
PSMA/FDG MTV	0.960 (0.601–1.417)	0.828		
PSMA/FDG TLU	1.035 (0.828–1.318)	0.746		
Dichotomized variables are indicated in the corresponding row				

*FDG* fluorodeoxyglucose, *MTV* metabolic tumor volume, *PRLT* prostate-specific membrane antigen targeted radioligand therapy, *PSA* prostate-specific antigen, *PSMA* prostate-specific membrane antigen, *SUV*_max_ maximum standardized uptake value, *SUV*_mean_ mean standardized uptake value, *TLU* total lesion uptake

ROC analysis identified a whole-body [^68^Ga]Ga-PSMA SUV_mean_ cutoff value of 10.23 as the best predictor of PSA30 response with an area under the curve (AUC) of 0.741 (95% CI 0.524–0.959, *p* = 0.030), while the optimal cutoff for the [^68^Ga]Ga-PSMA/[^18^F]FDG SUV_mean_ ratio was 2.08 (AUC 0.727, 95% CI 0.507–0.947, *p* = 0.043). ROC curves are reported in Supplementary Fig. 2. Interestingly, 12/13 (92%) of the non-responder patients had a [^68^Ga]Ga-PSMA SUV_mean_ <10 and all of them (13/13, 100%) had a [^68^Ga]Ga-PSMA/[^18^F]FDG SUV_mean_ ratio < 2.

### PSA50 response

PSA50 response was achieved in 9/25 (36%) [^18^F]FDG-positive patients, after a median time from the first PRLT administration of 84 days (range: 43–123 days). No imaging-derived parameters were associated with PSA50 response. At univariate analysis, only white blood cell count (WBC) was significantly associated with PSA50 response. WBC and time from diagnosis to PRLT were independent predictors of PSA50 at multivariate analysis. Full results are shown in Supplementary Table 2.

## Outcome analysis

**Fig. 4:**
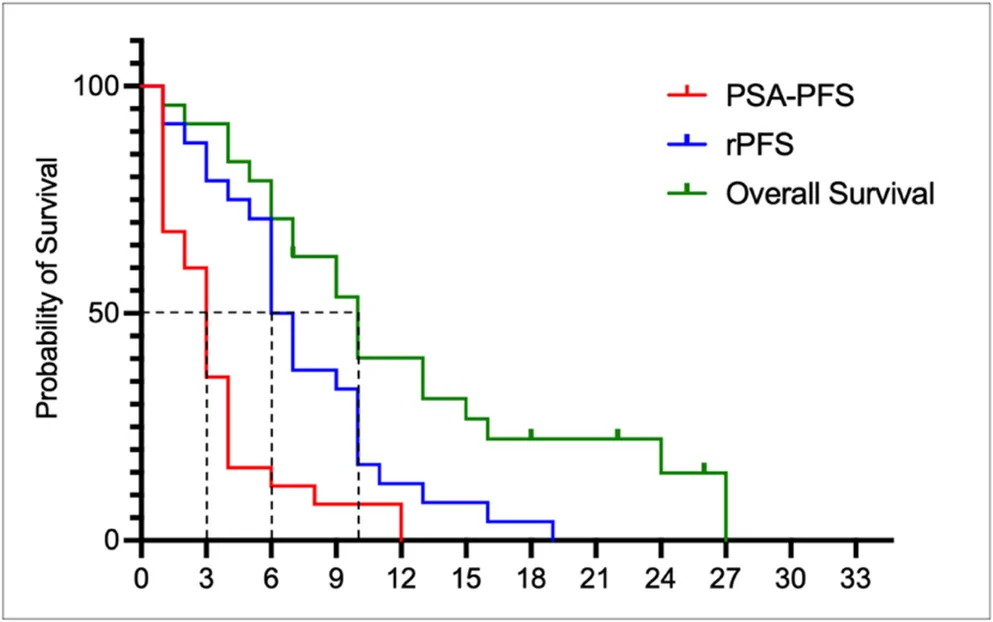
Kaplan-Meier analyses of treatment outcomes. The figure shows Kaplan–Meier curves of all three major treatment outcomes, i.e. prostate-specific antigen progression-free survival (PSA-PFS), radiographic progression-free survival (rPFS), and overall survival (OS). Tick marks on the curves indicate censored observations.

Figure 4 summarizes Kaplan–Meier survival curves of main treatment outcomes in our cohort

Median follow-up time was 11 months (range: 2–28 months) from the first PRLT cycle. Median PSA-PFS was 3 months (95%CI 2.1–3.9) following the first treatment cycle. None of the analyzed factors appeared to be correlated to PSA-PFS (data not shown).

**Fig. 5:**
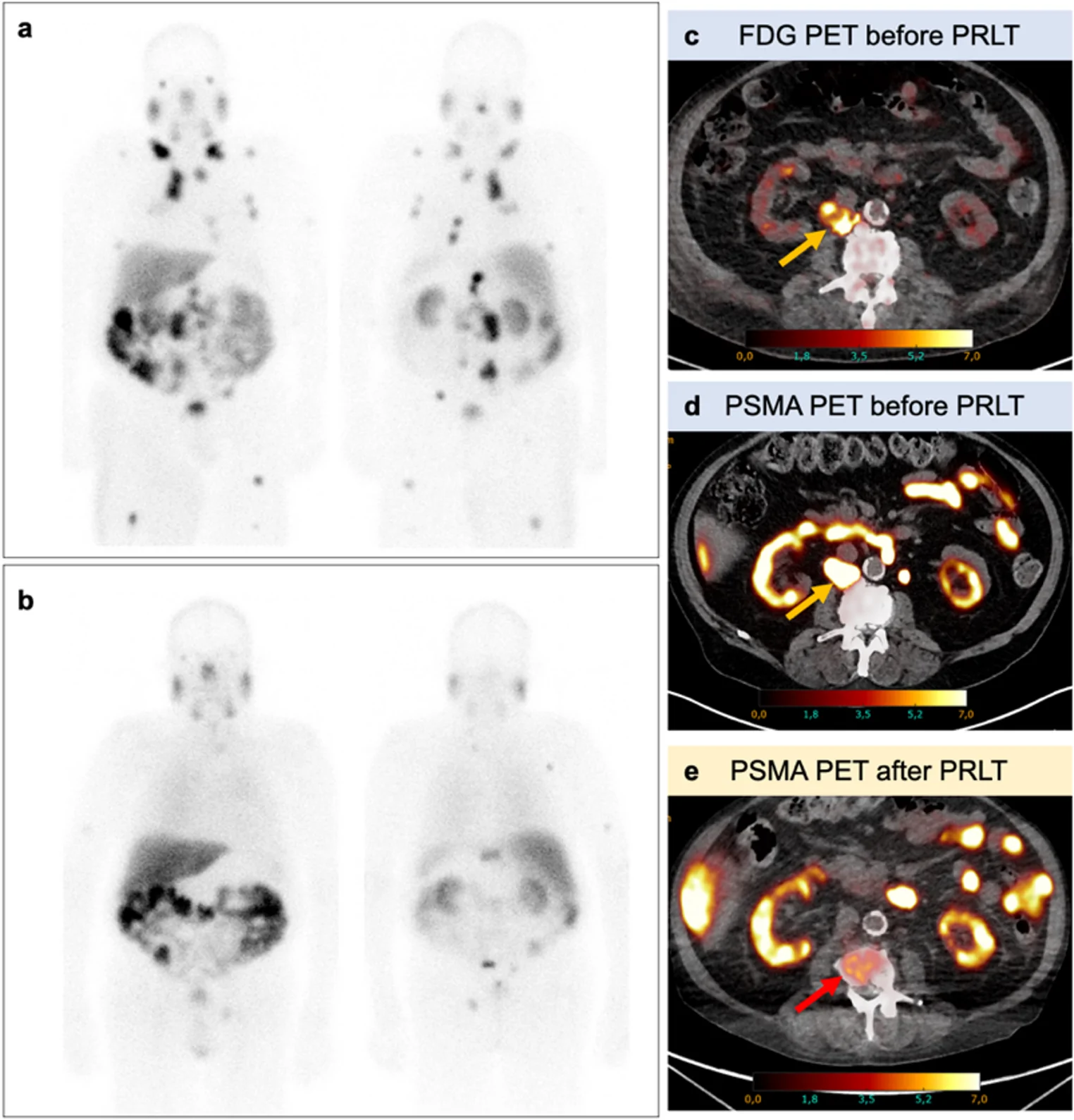
Mixed response to [^177^Lu]Lu-PSMA-I&T. Post-treatment [^177^Lu]Lu-PSMA-I&T scan showed a clear decrease of PSMA uptake from the 1 st (**a**) to the 4th (**b**) treatment cycle of the known sites of metastases, as well as the appearance of new lesions. Pre-treatment dual tracer PET/CTs demonstrate extensive metastatic disease involving lymph nodes (**c**-**d**, yellow arrows) and bone. Post-treatment [^68^Ga]Ga-PSMA-11 PET reveals an overall reduction in total disease burden, yet with new active bone lesions (**e**, red arrow), consistent with a mixed response. PSA levels decreased from 8.61 ng/mL before PRLT to 0.894 ng/mL, corresponding to a percentage reduction of 90%

Imaging restaging, scheduled two months after the last treatment cycle, was available for 13 patients. Ten patients were already deceased before restaging. One patient died 13 months following the first treatment cycle, without undergoing imaging restaging. One patient, in biochemical progression following the second treatment cycle, was lost at follow up. According to RECIST 1.1 and/or PPP criteria, most patients (*n* = 12) exhibited progressive disease. Of these 12 patients classified as having progressive disease by standardized criteria, two experienced a mixed response, with the appearance of more than two new lesions despite clear regression of some existing lesions (Fig. 5).

One patient showed stable disease with later progression 19 months following start of treatment.

Median rPFS was 6 months (95%CI 4.8–7.2). In the univariate Cox regression analysis, high ALP (HR 1.008, 95% CI 1.001–1.015; *p* = 0.023), [^68^Ga]Ga-PSMA-11 MTV (HR 1.007, 95% CI 1.002–1.012; *p* = 0.011), [^68^Ga]Ga-PSMA-11 TLU (HR 1.009, 95% CI 1.002–1.016; *p* = 0.015), and [^18^F]FDG MTV (HR 1.012, 95% CI 1.002–1.021; *p* = 0.013) were significantly associated with shorter rPFS. In the multivariate analysis, [^68^Ga]Ga-PSMA-11 MTV showed a trend towards a significant association with rPFS (HR 1.006, 95% CI 1.000–1.012; *p* = 0.061).

Median OS for [^18^F]FDG-positive patients was 10 months (95% CI 7.3–12.7). At the univariate Cox regression analysis, hemoglobin levels (HR 0.953, 95%CI, 0.919–0.987, *p* = 0.008), ALP (HR 1.010, 95%CI 1.002–1.019, *p* = 0.018) and PSA (HR 1.012, 95%CI 1.002–1.023, *p =* 0.023) levels before the start of treatment were associated with OS. Regarding imaging-based factors, [^68^Ga]Ga-PSMA-11 MTV (HR 1.009, 95%CI 1.003–1.015 *p* = 0.003), [^68^Ga]Ga-PSMA-11 TLU (HR 1.008, 95%CI 1.001–1.016, *p* = 0.025), and [^18^F]FDG MTV (HR 1.012, 95%CI 1.002–1.023, *p* = 0.024) were associated with OS. However, none of the variables remained significant in the multivariate model. Supplementary Tables 3 and Table 4 shows the results of the univariate and multivariate Cox regression analyses.

A secondary comparative analysis of response and survival outcomes between [^18^F]FDG-positive and [^18^F]FDG-negative patients is provided in the Supplementary Tables 5 and Supplementary Fig. 3.

## Toxicity

No high-grade hematological or renal toxicities (> Grade 2, CTCAE v5.0) were recorded during the observation period. In one patient who presented with Grade 2 renal toxicity prior to treatment, renal function remained stable during therapy, with serum creatinine levels of 2.3 mg/dL at baseline and 2.55 mg/dL at the last available measurement.

## Discussion

Over the last decade, [^177^Lu]Lu-PSMA therapy has emerged as a reliable treatment option for mCRPC patients. Currently, PSMA expression assessed by pre-treatment PSMA-targeted PET/CT remains the key eligibility criterion for patient selection. Nevertheless, a considerable proportion of patients (~ 50%) fail to respond to PRLT [1]. In this context, inter-patient and inter-tumor heterogeneity of PSMA expression represent unresolved issues that may affect the efficacy of PRLT [22, 23]. Consequently, there is a clear need to identify predictors of response to improve patient selection and ensure appropriate allocation of healthcare resources. It has been reported that up to 90% of patients with late-stage mCRPC present with at least one [^18^F]FDG-positive lesion [24–26], indicating that a substantial proportion of patients undergoing PRLT presents with at least one [^18^F]FDG-positive lesion before therapy. However, to the best of our knowledge, there are currently no data providing a clear view on response to PRLT in [^18^F]FDG-positive patients. Indeed, most of the available studies focused on the role of mismatch lesions [8, 27] or provided lesion-based analyses after 2 cycles of treatment only [28, 29], without explicitly reporting the overall outcomes of these patients. In large prospective trials such as the TheraP study [2, 12], as well as in other retrospective studies [30], patients with mismatch lesions were excluded. Furthermore, patients with concordant FDG+/PSMA+ lesions were analyzed together with patients with PSMA+/FDG− disease, although these subgroups are known to have different prognostic profiles. 

In our preliminary analysis we demonstrated that [^18^F]FDG-negative patients more frequently presented with exclusively nodal disease compared with [^18^F]FDG-positive ones (see supplementary data). This finding may suggest that patients with predominantly nodal involvement may harbor more differentiated tumors with less aggressive biological behavior than those with predominant bone disease, potentially explaining better responses to PRLT reported by previous studies [31–33]. We further observed that patients with [^18^F]FDG-positive disease had a higher number of prior lines of therapy, including chemotherapy, compared with [^18^F]FDG-negative patients, supporting the hypothesis that therapeutic pressure may select more aggressive tumor clones. Furthermore, we found a significant difference in both biochemical and radiologic response to PRLT between [^18^F]FDG-positive and [^18^F]FDG-negative patients (see supplementary data).

In this study, we investigated on potential predictors of response to [^177^Lu]Lu-PSMA-I&T in a rather homogeneous cohort of patients with [^18^F]FDG-positive mCRPC.

We found that [^68^Ga]Ga-PSMA-11 SUV_mean_ and [^68^Ga]Ga-PSMA/[^18^F]FDG SUV_mean_ ratio were the only significant predictors of PSA30 response. This demonstrates that [^68^Ga]Ga-PSMA-11 SUV_mean_ is a relevant independent predictor of response even in a population of [^18^F]FDG-positive patients. Our best-discriminating threshold value (SUV_mean_ 10.23) aligns closely with the cutoff used in the TheraP study (i.e., SUV_mean_ 10), suggesting that this SUV_mean_ cutoff value may serve as a clinically meaningful parameter for selecting candidates most likely to achieve a biochemical response to PRLT [12]. Similar findings have been reported by Telli et al., where PSMA SUV_mean_ was the sole significant predictor of PSA decline ≥ 50% in 152 patients receiving PRLT [30].

The cut-off value identified for [^68^Ga]Ga-PSMA/[^18^F]FDG SUV_mean_ ratio deserves a dedicated discussion. From a biological perspective, a higher PSMA-to-FDG uptake ratio may reflect a tumor phenotype in which PSMA expression predominates over metabolic activity, potentially identifying disease that remains more dependent on the PSMA pathway and therefore more amenable to PSMA-targeted radioligand therapy. Consistent with this interpretation, there were no significant correlations between uptake-based parameters (SUV_max_ and SUV_mean_) derived from [^68^Ga]Ga-PSMA-11 and [^18^F]FDG PET, as the two tracers capture distinct biological processes. While PSMA uptake reflects target expression, [^18^F]FDG uptake is more closely associated with tumor metabolic activity and dedifferentiation. Interestingly, all patients who did not achieve at least a 30% reduction in PSA had a PSMA-to-FDG mean uptake ratio below 2. The integration of these parameters may therefore provide complementary insights into tumor heterogeneity and biological aggressiveness. To the best of our knowledge, this ratio has not previously been reported and may represent a potentially useful metric for patient selection for PRLT, particularly among [^18^F]FDG-positive disease.

The analysis of the PSA50 response did not confirm the predictive value of either [^68^Ga]Ga-PSMA-11 SUV_mean_ or [^68^Ga]Ga-PSMA/[^18^F]FDG SUV_mean_ ratio, however this analysis was limited by the low number of events, which is compatible with the limited efficacy of PRLT in this heavily pretreated population. Previous similar cohorts of mCRPC patients used PSA30 rather than PSA50 threshold as a measure of outcome [34–37], since a stronger correlation with OS was found for PSA30 compared to the more conventional PSA50 threshold [38–41].

However, it remains questionable whether a PSA decline without a response at the imaging assessment should be considered as a clinically meaningful response in this setting of patients with rapidly progressing disease and poor prognosis. In fact, despite the reduction in PSA levels, radiological assessment demonstrated high rates of disease progression during or shortly after therapy (98%), indicating that [^18^F]FDG-positive patients generally do not experience sustained long-term benefit. Of note, we also noticed that in cases of mixed radiological response, current standardized criteria may not always reflect biochemical response. This observation suggests a nuanced understanding of “progression”, where minor radiologic worsening may still coexist with effective antitumor activity [42]. Patients may achieve temporary disease control when PSMA expression remains sufficient to enable effective targeting of at least part of the tumor burden, as illustrated by lesions that respond to therapy despite exhibiting positive [^18^F]FDG uptake (Fig. 5). This observation warrants further investigation in larger studies, particularly for patients lacking alternative therapeutic options, and suggests that combined therapeutic approaches may be explored in this subgroup.

Concerning prognostic biomarkers of rPFS and OS, ALP demonstrated a significant association with both rPFS and OS, whereas hemoglobin levels and PSA levels were associated with OS. With respect to imaging-based parameters, [^68^Ga]Ga-PSMA-11 MTV, [^68^Ga]Ga-PSMA-11 TLU, and [^18^F]FDG MTV were associated with both rPFS and OS, confirming the role of tumor burden as a key determinant of survival [12, 43–45]. Although the hazard ratios and the confidence intervals require cautious interpretation of the results, the identified prognostic parameters for survival outcomes are in line with previously reported data from larger patient cohorts [6, 7, 19]. In a sub-analysis of the TheraP trial, Buteau et al. [12] found that high [^18^F]FDG MTV (≥ 200 mL) was associated with a lower PSA response, regardless of treatment, confirming that [^18^F]FDG MTV has prognostic value but it does not appear as a predictor of response to PRLT. In our population as well, [^18^F]FDG MTV was associated with prognosis but was not predictive of PSA response to PRLT. However, despite the trend shown by [^68^Ga]Ga-PSMA-11 MTV in predicting rPFS, the lack of significant associations between imaging parameters and survival outcomes in multivariate analyses may also suggest that these imaging parameters do not provide additional prognostic information over established biochemical factors related to tumor burden (e.g., hemoglobin, ALP, PSA). Nevertheless, although biologically plausible in this advanced disease setting, this finding should be interpreted with caution given the limited sample size of our cohort.

This study has several limitations that should be acknowledged. First, its retrospective design and the relatively small sample size limit the generalizability of our findings and the statistical power of the analyses despite the use of penalized methods and the strategies used to avoid overfitting. Yet, the purpose of this study was to report the real-world clinical experience of a single center in a specific clinical scenario that has been only marginally explored in the literature, particularly in a setting where dual-tracer PET/CT is not routinely adopted in clinical practice. Another limitation is represented by the absence of dosimetric evaluation that prevented the assessment of tumor-absorbed dose, which is known to correlate with treatment response [46] and may represent a key variable to be considered in future studies [47]. The availability of dosimetric data could contribute to a more accurate understanding of the actual effectiveness of PRLT in this subgroup of patients with unfavorable outcomes. Moreover, the low proportion of patients with mismatch lesions (8%) precluded any meaningful subgroup or exploratory analysis. While no definitive conclusions can be drawn regarding the clinical significance of mismatch lesions in our cohort of [^18^F]FDG-positive patients, previous evidence suggests that among patients considered eligible for PSMA-targeted therapy, the impact of additional FDG/PSMA mismatch lesions is generally low, accounting for only a small proportion of cases (< 5%) [48]. Finally, the lack of post-treatment [^18^F]FDG PET/CT restaging did not allow an assessment of changes in tumor glucose metabolism possibly induced by PRLT.

## Conclusions

Our findings in a cohort of [^18^F]FDG-positive mCRPC patients treated with [^177^Lu]Lu-PSMA-I&T underscore the role of [^68^Ga]Ga-PSMA-11 PET metrics in selecting candidates for PRLT. The [^68^Ga]Ga-PSMA-11/[^18^F]FDG SUV_mean_ ratio was also identified as a new predictor of biochemical response in these group of patients. In particular, a whole-body [^68^Ga]Ga-PSMA-11 SUV_mean_ higher than 10 and a [^68^Ga]Ga-PSMA-11/[^18^F]FDG SUV_mean_ ratio > 2 may help identify patients likely to achieve at least a 30% reduction in PSA levels, even among [^18^F]FDG-positive mCRPC patients. In addition, our study further supports the prognostic significance of tumor burden either evaluated by imaging-based volumetric parameters or by known biochemical markers of disease. [^18^F]FDG was suggested as a possible prognostic biomarker of survival, rather than a predictor of response to PRLT. Further larger studies in homogeneous cohorts of patients with [^18^F]FDG-positive mCRPC are needed to confirm our preliminary results.

## Supplementary Information

Below is the link to the electronic supplementary material.


Supplementary Material 1 (DOCX 376 KB)


## Data Availability

The datasets generated during and/or analyzed during the current study are available from the corresponding author on reasonable request.
