# Supplementary material for: Real-world outcomes of [177Lu]Lu-PSMA-I&T in [18F]FDG-positive metastatic castration-resistant prostate cancer: factors related to response and survival
Source: Eur J Nucl Med Mol Imaging. 2026 Jun 6;53(11):6351–62. doi: 10.1007/s00259-026-07972-6 (PMC13526135; doi:10.1007/s00259-026-07972-6)
Supplement: Supplementary file 1 [file 259_2026_7972_MOESM1_ESM.docx]

**Supplementary data**

**Supplementary Table 1.** Patient Characteristics of [^18^F]FDG-negative patients (*n* = 9)

| Parameter | Value |
| --- | --- |
| Age at C1 (years) | 74 (54 – 93) |
| ISUP grade  1  2  4  5  Missing | 1 (11)  2 (22)  2 (22)  3 (34)  1 (11) |
| Prior treatment  Docetaxel  Cabazitaxel  Enzalutamide  History of  Abiraterone  History of  Ongoing  ^223^RaCl_2_ | 4 (44)  2 (22)  2 (22)  4 (44)  2 (22)  1 (11) |
| Laboratory data before PRLT  PSA (ng/ml)  Hb (g/L)  White cell count (10^9^/L)  Platelet count (10^9^/L)  Creatinine (mg/dL)  Alkaline phosphatase (U/l)  Albumin (g/L) | 10.5 (0.39 – 85.70)  12.5 (11.0 – 15.4)  8.5 (4.0 – 10.50)  247 (151 – 353)  0.93 (0.66 – 1.32)  67 (58.5 – 283)  3805 (3178 – 4158) |
| Site of metastases before PRLT  Bone  Exclusive bone disease  Lymph node  Exclusive lymph node disease  Visceral (Liver) | 5 (56)  2 (22)  7 (78)  4 (44)  1 (11) |
| For continuous variables median values (range) are reported.  For categorical variables, number of patients (percent) are reported. | |

Notes: C1 = 1^st^ PRLT cycle; Hb = hemoglobin; PRLT = prostate-specific membrane antigen targeted radioligand therapy; PSA = prostate specific antigen.

**Supplementary Table 2.** Univariate and multivariate logistic regression analysis for PSA50 response

|  | **Univariate** | | **Multivariate** | |
| --- | --- | --- | --- | --- |
| **Variable** | Odd ratio (95%CI) | *p* | Odd ratio (95%CI) | *p* |
| **Clinical factors** | | | | |
| Time from diagnosis to PRLT | 1.007 (0.998 - 1.019) | 0.141 | **1.012 (1.001 – 1.029)** | **0.038** |
| Bone mets (no vs. yes) | 1.050 (0.118 - 12.939) | 0.966 |  |  |
| Lymph node mets (no vs. yes) | 0.727 (0.130 - 4.187) | 0.713 |  |  |
| Visceral mets (no vs. yes) | 0.852 (0.122 - 5.016) | 0.860 |  |  |
| Chemotherapy (yes vs. no) | 1.400 (0.184 - 16.525) | 0.752 |  |  |
| N° previous lines (<2 vs. ≥2) | 0.517 (0.037 – 7.213) | 0.597 |  |  |
| **Biochemical factors** | | | | |
| **White cell count (10^9^/L)** | **1.771 (1.029 - 3.755)** | **0.038** | **2.738 (1.263 – 9.543)** | **0.006** |
| Hemoglobin (g/L) | 1.040 (0.986 - 1.110) | 0.155 |  |  |
| Platelet count (10^9^/L) | 1.000 (0.989 - 1.011) | 0.975 |  |  |
| Alkaline phosphatase (U/L) | 1.004 (0.995 - 1.022) | 0.325 |  |  |
| PSA (ng/mL) | 0.999 (0.978 - 1.020) | 0.994 |  |  |
| **Imaging-based factors** | | | | |
| PSMA SUV_max_ | 1.008 (0.978 - 1.039) | 0.602 |  |  |
| PSMA SUV_mean_ | 1.118 (0.867 - 1.483) | 0.389 |  |  |
| PSMA MTV | 1.002 (0.993 - 1.012) | 0.588 |  |  |
| PSMA TLU | 1.003 (0.991 - 1.016) | 0.570 |  |  |
| FDG SUV_max_ | 0.977 (0.851 - 1.091) | 0.696 |  |  |
| FDG SUV_mean_ | 1.163 (0.658 - 2.173) | 0.578 |  |  |
| FDG MTV | 1.004 (0.991 - 1.023) | 0.470 |  |  |
| FDG TLU | 1.012 (0.982 - 1.052) | 0.403 |  |  |
| PSMA/FDG SUV_max_ | 1.135 (0.804 - 1.624) | 0.464 |  |  |
| PSMA/FDG SUV_mean_ | 1.305 (0.445 - 3.918) | 0.616 |  |  |
| PSMA/FDG MTV | 0.989 (0.606 - 1.460) | 0.954 |  |  |
| PSMA/FDG TLU | 0.988 (0.758 - 1.233) | 0.917 |  |  |
| Dichotomized variables are indicated in the corresponding row | | |  |  |

Notes: FDG = fluorodeoxyglucose; MTV = metabolic tumor volume; PRLT = prostate-specific membrane antigen targeted radioligand therapy; PSA = prostate-specific antigen; PSMA = prostate-specific membrane antigen; SUVmax = maximum standardized uptake value; SUVmean = mean standardized uptake value; TLU = total lesion uptake.

**Supplementary Table 3.** Univariate and multivariate Cox regression analysis for rPFS

|  | **Univariate** | | **Multivariate** | |
| --- | --- | --- | --- | --- |
| **Variable** | Hazard ratio (95%CI) | *p* | Hazard ratio (95%CI) | *p* |
| Clinical factors | | | | |
| Time from diagnosis to PRLT | 0.995 (0.989–1.001) | 0.056 |  |  |
| Bone mets (no vs. yes) | 1.097 (0.319–3.772) | 0.974 |  |  |
| Lymph node mets (no vs. yes) | 2.313 (0.892–6.002) | 0.085 |  |  |
| Visceral mets (no vs. yes) | 1.287 (0.518–3.198) | 0.587 |  |  |
| Chemotherapy (no vs. yes) | 0.445 (0.138–1.435) | 0.175 |  |  |
| N° previous lines (<2 vs. ≥2) | 0.444 (0.099–1.995) | 0.290 |  |  |
| Biochemical factors | | |  |  |
| White cell count (10^9^/L) | 0.949 (0.747–1.206) | 0.669 |  |  |
| Hemoglobin (g/L) | 0.979 (0.952–1.007) | 0.142 |  |  |
| Platelet count (10^9^/L) | 0.995 (0.989–1.002) | 0.163 |  |  |
| **Alkaline phosphatase (U/L)** | **1.008 (1.001–1.015)** | **0.023** | 1.006 (0.999 – 1.012) | 0.499 |
| PSA (ng/mL) | 1.006 (0.996–1.015) | 0.241 |  |  |
| Imaging-based factors | | |  |  |
| PSMA SUV_max_ | 1.002 (0.985–1.020) | 0.799 |  |  |
| PSMA SUV_mean_ | 0.965 (0.842–1.106) | 0.614 |  |  |
| **PSMA MTV** | **1.007 (1.002–1.012)** | **0.011** | 1.006 (1.000-1.012) | 0.061 |
| **PSMA TLU** | **1.009 (1.002–1.016)** | **0.015** |  |  |
| FDG SUV_max_ | 0.994 (0.931–1.062) | 0.863 |  |  |
| FDG SUV_mean_ | 0.876 (0.596–1.286) | 0.499 |  |  |
| **FDG MTV** | **1.012 (1.002–1.021)** | **0.013** |  |  |
| FDG TLU | 1.016 (0.997–1.035) | 0.104 |  |  |
| Dichotomized variables are indicated in the corresponding row | | | | |

Notes: FDG = fluorodeoxyglucose; MTV = metabolic tumor volume; PRLT = prostate-specific membrane antigen targeted radioligand therapy; PSA = prostate-specific antigen; PSMA = prostate-specific membrane antigen; SUV_max_ = maximum standardized uptake value; SUV_mean_ = mean standardized uptake value; TLU = total lesion uptake.

**Supplementary Table 4.** Univariate and multivariate Cox regression analysis for overall survival

|  | **Univariate** | | **Multivariate** | |
| --- | --- | --- | --- | --- |
| **Variable** | Hazard ratio (95%CI) | *p* | Hazard ratio (95%CI) | *p* |
| Clinical factors | | | | |
| Time from diagnosis to PRLT | 0.994 (0.988–1.001) | 0.100 |  |  |
| Bone mets (no vs. yes) | 1.025 (0.231–4.540) | 0.974 |  |  |
| Lymph node mets (no vs. yes) | 1.721 (0.609–4.863) | 0.305 |  |  |
| Visceral mets (no vs. yes) | 1.420 (0.495–4.078) | 0.515 |  |  |
| Chemotherapy (no vs. yes) | 0.463 (0.126–1.702) | 0.246 |  |  |
| N° previous lines (<2 vs. ≥2) | 0.208 (0.043–1.012) | 0.052 |  |  |
| Biochemical factors | | |  |  |
| White cell count (10^9^/L) | 1.049 (0.805–1.366) | 0.724 |  |  |
| Hemoglobin (g/L) | **0.953 (0.919–0.987)** | **0.008** | 0.968 (0.928–1.010) | 0.130 |
| Platelet count (10^9^/L) | 1.000 (0.993–1.007) | 0.979 |  |  |
| Alkaline phosphatase (U/L) | **1.010 (1.002–1.019)** | **0.018** |  |  |
| PSA (ng/mL) | **1.012 (1.002–1.023)** | **0.023** |  |  |
| Imaging-based factors | | |  |  |
| PSMA SUV_max_ | 0.999 (0.982–1.017) | 0.950 |  |  |
| PSMA SUV_mean_ | 0.973 (0.840–1.126) | 0.709 |  |  |
| PSMA MTV | **1.009 (1.003–1.015)** | **0.003** | 1.005 (0.998–1.013) | 0.166 |
| PSMA TLU | **1.008 (1.001–1.016)** | **0.025** |  |  |
| FDG SUV_max_ | 1.005 (0.930–1.086) | 0.900 |  |  |
| FDG SUV_mean_ | 0.931 (0.642–1.349) | 0.704 |  |  |
| FDG MTV | **1.012 (1.002–1.023)** | **0.024** |  |  |
| FDG TLU | 1.017 (0.995–1.039) | 0.124 |  |  |
| Dichotomized variables are indicated in the corresponding row | | |  |  |

Notes: FDG = fluorodeoxyglucose; MTV = metabolic tumor volume; PRLT = prostate-specific membrane antigen targeted radioligand therapy; PSA = prostate-specific antigen; PSMA = prostate-specific membrane antigen; SUV_max_ = maximum standardized uptake value; SUV_mean_ = mean standardized uptake value; TLU = total lesion uptake.

**Supplementary Table 5.** Comparison of PSA and imaging response between [^18^F]FDG-positive and [^18^F]FDG-negative patients.

|  | [^18^F]FDG-positive | [^18^F]FDG-negative | *p* |
| --- | --- | --- | --- |
| PSA50 response | 9/25 (36%) | 8/9 (89%) | **0.007** |
| PSA30 response | 11/25 (44%) | 8/9 (89%) | **0.02** |
| Imaging complete/partial response at three months* | 0/13 (0%) | 4/8 (50%) | **0.005** |

*Of the available.

**Supplementary Figure 1.** Clinical factors associated with [^18^F]FDG-positivity. a) [^18^F]FDG-negative patients were more likely to have exclusive lymph node involvement compared to [^18^F]FDG-positive patients (44% vs. 12%, *p* = 0.039; X^2^ test). Conversely, b) [^18^F]FDG positive patients were more likely to have more than two previous lines of treatment (92% vs. 44%, *p* = 0.003; X^2^ test), c) including chemotherapy (83% vs. 17%, *p* = 0.025; X^2^ test). Of note, [^18^F]FDG-positive patients also exhibited lower hemoglobin levels (111.6 ± 16.8 vs. 125.6 ± 13.5 g/L, *p* = 0.032, t-test) and lower white blood cell counts (6.13 ± 1.56 vs. 7.70 ± 2.26 × 10⁹/L, *p* = 0.029, t-test) than [^18^F]FDG-negative patients before PRLT initiation. No other clinical or biochemical variables were significantly associated with [^18^F]FDG positivity (data not shown).

**Supplementary Figure 2.** Receiver operating characteristic (ROC) curves evaluating the ability of [^68^Ga]Ga-PSMA SUV_mean_ and the [^68^Ga]Ga-PSMA/[^18^F]FDG SUV_mean_ ratio to predict PSA30 response.

**Supplementary Figure 3.** Kaplan–Meier curves comparing progression-free survival (rPFS, left panel) and overall survival (OS, right panel) between [^18^F]FDG-positive and [^18^F]FDG-negative patients. [^18^F]FDG-negative patients showed significantly longer rPFS (*p* = 0.027, log-rank test) and OS (*p* = 0.016, log-rank test) than [^18^F]FDG-positive patients.
